# Self-Assembled H-Bonding Superstructures for Alkali Cation and Proton Transport

**DOI:** 10.3389/fchem.2021.678962

**Published:** 2021-05-06

**Authors:** Erol Licsandru, Iuliana-Marilena Andrei, Arie van der Lee, Mihail Barboiu

**Affiliations:** Institut Europeen des Membranes, University of Montpellier, ENSCM-CNRS, Montpellier, France

**Keywords:** ion-channels, bilayer-membranes, proton transport, self-assembly, H-bonding

## Abstract

Transmembrane protein channels are of significant importance for the design of biomimetic artificial ion channels. Regarding the transport principles, they may be constructed from amphiphilic compounds undergoing self-assembly that synergistically generate directional superstructures across bilayer membranes. Particularly interesting, these alignments may impose an artificial pore structure that may control the ionic conduction and translocate water and ions sharing one pathway across the cell membrane. Herein, we report that the imidazole and 3-amino-triazole amphiphiles self-assemble *via* multiple H-bonding to form stable artificial networks within lipid bilayers. The alignment of supramolecular assemblies influences the conduction of ions, envisioned to diffuse along the hydrophilic pathways. Compounds **1-8** present subtle variations on the ion transport activities, depending the structure of hydrophilic head and hydrophobic components. Fluorinated compounds **3**, **4** and **7**, **8** outperform the corresponding non-fluorinated counterparts **1**, **2** and **5**, **6**. Under the same conditions, the R enantiomers present a higher activity vs. the S enantiomers. The present systems associating supramolecular self-assembly with ion-transport behaviors may represent very promising unexplored alternatives for ion-transport along with their transient superstructures within bilayer membranes, paralleling to that of biology.

## Introduction

Ion transport across the cell membrane is a highly important physiological process. Transmembrane protein channels, able to translocate cations across lipid bilayer with high permeability and selectivity (Lehninger et al., 2005). Inspired by the natural protein channels, synthetic artificial ion channels or carriers are fabricated as simplified models to study the driving factors for translocation and for their potential use as drugs, sensors or active membrane components. Synthetic ion channels are simple molecules, constructed by combining hydrophobic and hydrophilic groups. The hydrophobic part is required in order to have a good affinity with the lipid bilayer membrane, while the hydrophilic part offers the possibility to interact with the ions or other hydrophilic metabolites, either in their hydrated or in their dehydrated forms (Hille, 2001). There are two strategies to build synthetic ion channels: (a) the unimolecular channels are large molecules which are long enough to entirely cross the lipid bilayer membrane; they contain a lipophilic exterior and polar hydrophilic interior and (b) the self-assembled channels which are superstructures resulting from the self- assembly of a number of building blocks. The self-assembled channels having better solubility, allow a larger amount of active compound to act on the substrate, compared to the unimolecular channels (Zheng et al., 2021).

We have shown in previous studies that the transport activity has an optimal relationship with lipophilicity of ionophores, while the disposition of the hydrophilic groups on the molecule' backbone proved to be important too for selective ion binding (Barboiu, 2004). The self-assembly of the functional transporting superstructures is directly determined by the directional self-assembly groups. The structural dynamics control the stability self-organized superstructures along cation recognition and transporting pathways. The transport mechanism is determined by the optimal coordination rather than classical dimensional compatibility between hydrophilic binding units and ions and systematic changes of the structure lead to adaptive selection in cation-transport activity (Valkenier et al., 2014).

**Scheme 1:**
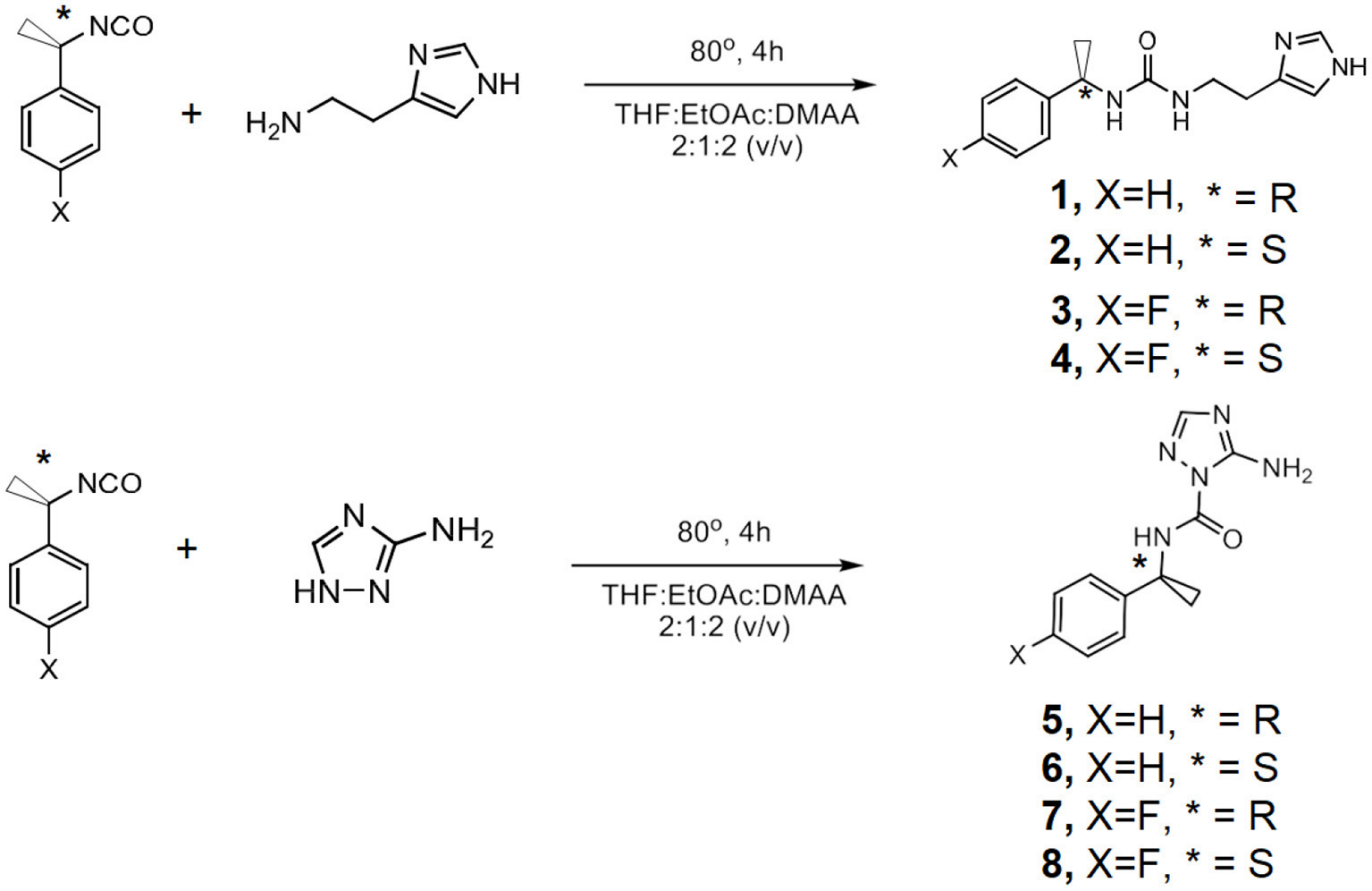
Synthetic route used in the synthesis of the compounds **1**-**8**

Herein, a library of eight heterocyclic compounds was considered for the bilayer membrane transport studies described here (Scheme 1; Supplementary Table 1). They present: (a) an aromatic chiral hydrophobic group acting as a membrane anchoring component; given the chirality of lipids, the assessment of the chirality influence on transport activity is important; (b) half of the compounds contain fluorine atoms grafted of the aromatic ring. It has been shown by Karagiannidis et al. (2014) that minor structural modifications on aromatic rings, in the form of fluorine atoms, has presented a significant influence on the activity in the chloride/nitrate antiport. Significant increase in anion transport activity with such a minor structural difference as a fluorine atom is that the modification of lipophilicity changes significantly the interaction with the lipid bilayer. Since the influence of this structural modification is tied to the lipid membrane, but was proven for antiport anion transporters, we aimed to reveal this influence of such a modification in the transport of cations; (c) a H-bonding urea/amide group as H-bonding directional self-assembling components; and (d) a heterocyclic hydrophilic head, namely imidazole and 3-aminotriazole for cation/proton binding and transport.

## Methods

### LUV Preparation

Egg yolk L-α-phosphatidylcholine (EYPC chloroform solution, 20 mg, 26 mmol) was dissolved in a 1:1 CHCl_3_/MeOH mixture (2 ml), the solution was evaporated under reduced pressure and the resulting thin film was dried under high vacuum for 2 h. The lipid film was hydrated in 0.4 mL of phosphate buffer (10 mM sodium phosphate, pH = 6.4, 100 mM NaCl) containing 10 μM HPTS (8-hydroxypyrene-1,3,6-trisulfonic acid trisodium salt) for 40 min. After hydration, the suspension was submitted to 5 freeze-thaw cycles (liquid nitrogen, water at room temperature). The large multilamellar liposome suspension (0.4 mL) was submitted to high-pressure extrusion at room temperature (21 extrusions through a 0.1 m polycarbonate membrane afforded a suspension of LUVs with an average diameter of 100 nm). The LUV suspension was separated from extravesicular dye by size exclusion chromatography (SEC) (stationary phase: Sephadex G-50, mobile phase: phosphate buffer) and diluted to 2.8 ml with the same phosphate buffer to give a stock solution with a lipid concentration of 3.66 mM (assuming 100% of lipid was incorporated into liposomes).

### Cation Transport Experiments

100 μL of HPTS-loaded vesicles (stock solution) was suspended in 1.9 mL of the buffer (PBS 10 mM pH=6.4 containing 100 mM of NaCl) and placed into a fluorimetric cell. The emission of HPTS at 510 nm was monitored with excitation wavelengths at 403 and 460 nm simultaneously. During the experiment, 20 μL of a 0-30 mM DMSO solution of the compound was added at t = 50 s, followed by injection of 21 μL of 0.5 M aqueous NaOH at t = 100 s. The addition of the NaOH resulted in a pH increase of ~1 pH unit in the extra vesicular buffer. Maximal possible changes in dye emission were obtained at t = 500 s by lysis of the liposomes with detergent (40 μL of 5% aqueous Triton X100). The experiment is ended at t = 800 s. This offers a window of 500 s of transport. The final transport trace was obtained as a ratio of the emission intensities monitored at 460 and 403 nm and normalized to 100% of transport. In the case of other cations, the protocol is adapted by changing the 100 mM NaCl in the PBS buffer with a solution of the respective cation.

### Proton Transport Experiments

100 μL of HPTS-loaded vesicles (stock solution) was suspended in 1.9 mL of the buffer (PBS 10 mM pH=6.4 containing 100 mM of KCl) and placed into a fluorimetric cell. The emission of HPTS at 510 nm was monitored with excitation wavelengths at 403 and 460 nm simultaneously. During the experiment, 20 μL of a 0-maximum allowed concentration mM DMSO solution of the compound of interest was added at t = 50s, followed by injection of 20 μL of 1 nM solution of valinomycin in DMSO at t = 100s. at time =150s an injection of 21 μL of 0.5 M aqueous NaOH. The addition of the NaOH resulted in a pH increase of ~1 pH unit in the extra vesicular buffer. Maximal possible changes in dye emission were obtained at t = 500s by lysis of the liposomes with detergent (40 μL of 5% aqueous Triton X100). The experiment is ended at t = 800s. This offers a window of 500 s of transport. The final transport trace was obtained as a ratio of the emission intensities monitored at 460 and 403 nm and normalized to 100% of transport. This offers a window of 500 s of transport. The final transport trace was obtained as a ratio of the emission intensities monitored at 460 and 403 nm and normalized to 100% of transport.

## Results And Discussions

Four R-benzylureido-ethylimidazoles, **1**-**4** and four R-benzyl-amido-2-(3-amino triazole) **5**-**8** (R=H, F) compounds have been prepared for the studies described here.

The (R)- or (S)-(1-isocyanatoethyl)-benzene, and (R)- or (S)-1-fluoro-4-(1-isocyanatoethyl)-benzene were treated with the corresponding histamine or the 3-amino-triazole (THF/EtOAc/ DMAC, 80°C, 15 min) to afford after precipitation from acetonitrile compounds **1**-**8**, respectively, as white powders (**Scheme 1**). We noted that electrophilic isocyanate group selectively reacted with N2-imidazole of 3-amino-triazole, which is more nucleophilic than -NH_2_ group. ^1^H-NMR and ESI-MS spectroscopic data are in accord with proposed structures (Supporting Information for details).

**Figure 1:**
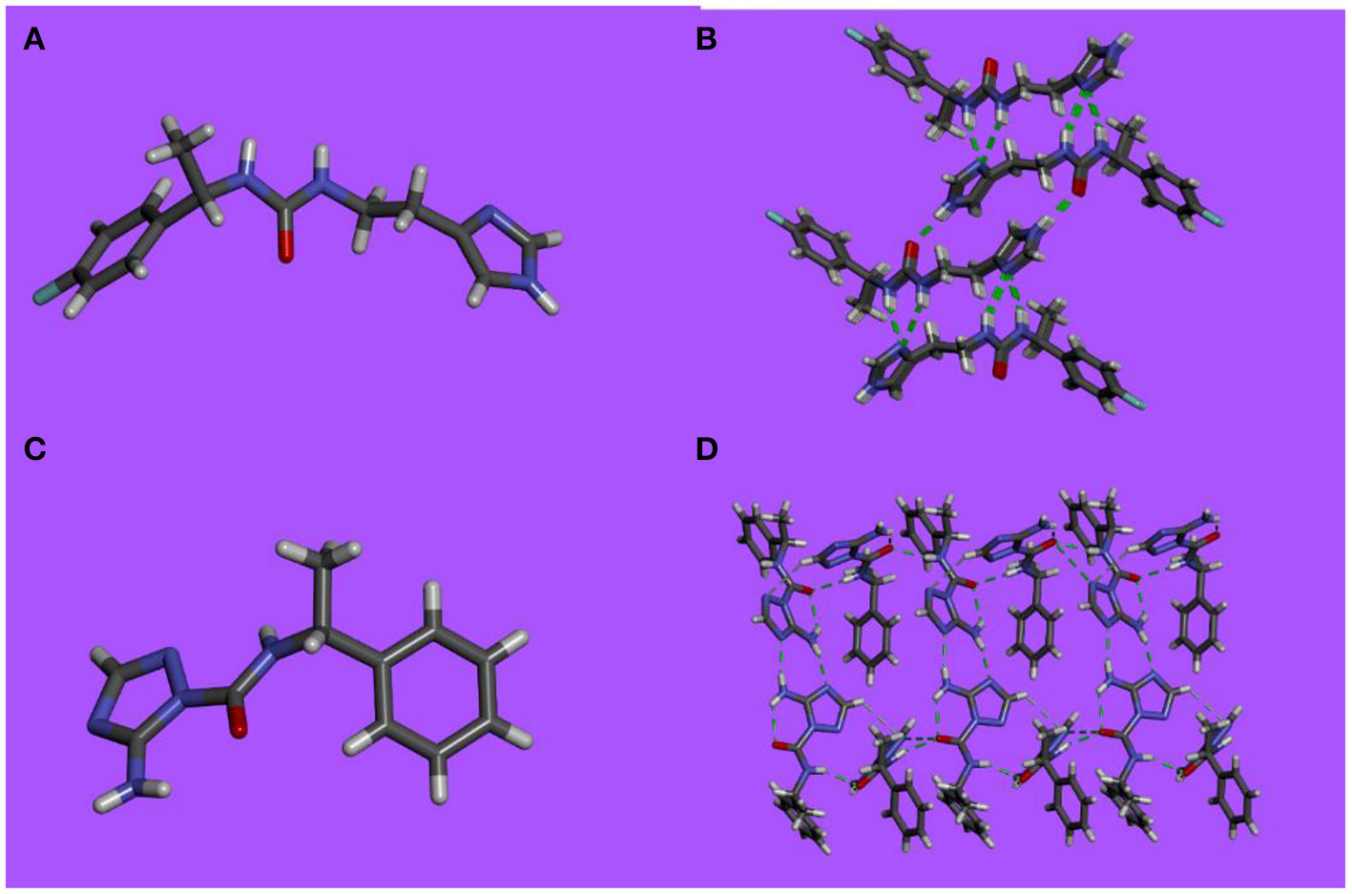
X-ray single-crystal structure and packing in stick representation of **(A,B) 3** and **(C,D) 5**.

Single-crystals suitable for X-ray analysis were obtained by slow evaporation of aqueous methanolic or ethanolic solutions of compounds **3**-**6** at room temperature. The X-ray analysis of the single-crystal packings reveal that these compounds undergo self-assembly *via* extended H-bonding π-π stacking, hydrophobic interactions and form networks that may provide information on the possible assemblies that the molecules can adopt in bilayers (Figure 1).

Analysis of the single-crystal packing of compounds **3** and **4** shows heteromeric exclusive H-bonding *via* head to tail associations between urea and imidazole groups (Figures 1A,B). The successive H-bonding sequences strongly enforce the directional winding of dimers allowing considerable overlap between the aromatic rings on vicinal hydrophobic layers. Supplementary hydrogen bonds by the fluorine atom toward two slightly acidic hydrogen atoms generates a distortion of the stacks as the aromatic rings are not in the same plane.

The single-crystal packing of compounds **5** and **6** display a very tight packing, as a consequence of multiple H-bonding (Figures 1A,B). They tend to assemble in a “head-to-head” conformation *via*- N_Im_···H_2_N H-bonds between the amino-triazole units on one side, while supplementary double H-bonds via – NHCO- motifs are formed with neighboring molecules on the other side. The phenyl ring is almost perpendicular to the 3-amino triazole moiety, with an angle of 83.4° between the triazole NH, the urea oxygen and the para hydrogen of the phenyl ring. The packing of compounds **5** and **6** have an embraced disposition, hydrophobic pockets being plugged by a perpendicularly oriented triazole moiety.

These directional oriented structures do present open channel type structures in solid state, which does not necessarily imply the inactivity of the compound in transport experiments. Through the presence of multiple weak bonds as well as an amphiphilic character allow possible interaction with hydrated ions.

**Figure 2:**
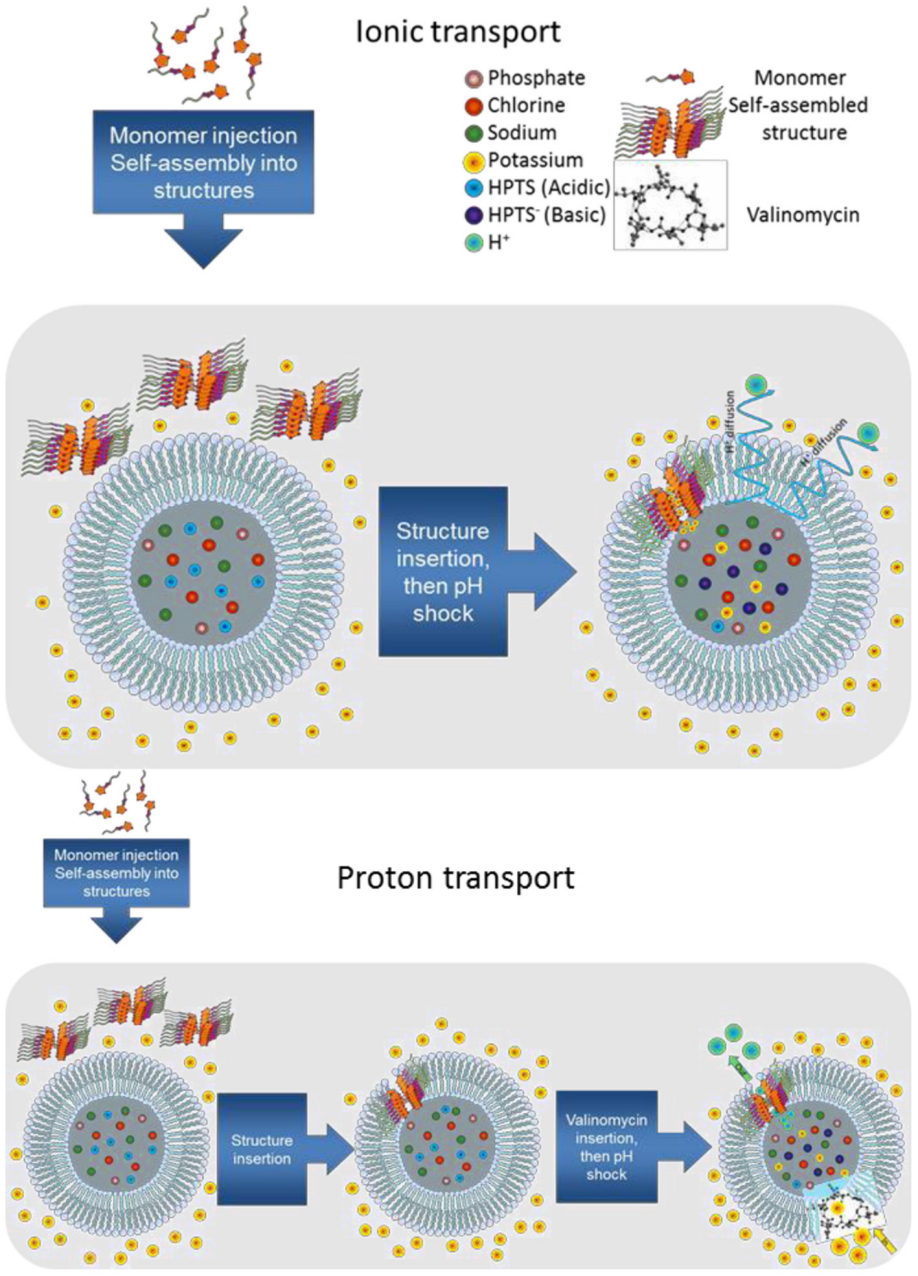
Schematic representations of ionic and proton HPTS transport assays.

The ion-transport activity for each compound was evaluated by HPTS assay (Berezin and Davis, 2009) (Figure 2). EYPC liposomes (Large Unilamellar Vesicles-LUV, 100 nm) were filled with a pH-sensitive dye, 8-Hydroxypyrene-1,3,6-trisulfonic acid trisodium salt (HPTS) and 100 mM NaCl in a phosphate buffer (10 mM, pH 6.4). The liposomes were then suspended in an external phosphate buffer (10 mM, pH 6.4) containing 100 mM of MCl, M^+^= Na^+^, K^+^. Then, after addition in the bilayer membrane *via* the injection of 20 μL of aliquots from stock 10 mM DMSO solutions of **1-8**, an external pH gradient was created by addition of NaOH. The internal pH change inside the liposome was monitored by the change in the fluorescence of HPTS over a period of 800 s, out of which, the active transport took place over 500 s. The resulting transport curves are therefore presented expressed in N_500_ in function of time (Supplementary Figures 1, 2).

Compounds **1-8** are presenting subtle variations on the transport activities of Na^+^ (Supplementary Figure 1) and K^+^ (Supplementary Figure 2), depending the structure of hydrophilic head and hydrophobic components. We note that fluorinated compounds **3**, **4** and **7**, **8** outperform the corresponding counterparts **1**, **2** and **5**, **6**, that are lacking the fluorine substituent. They have rather same N_500_ activity for translocation of K^+^ (low activity) and Na^+^ (fair to very good activity) at the same concentration, with the histamine derivatives especially performing an excellent transport (Table 1). Under the same conditions, the R enantiomers present a better activity vs. the S enantiomers with the exception of compound **4**, that is the most active of the library.

**Table 1:** Hill analysis results of Na^+^, K^+^ and H^+^ transport with compounds **1-8**, in absence (Na+, K+) or presence of Val (H+).

**Compound**	**1**	**2**	**3**	**4**	**5**	**6**	**7**	**8**
**Na**^**+**^								
N_max500_	0.50	0.50	0.72	1	0.52	NA	0.78	0.60
Activity	Weak	Weak	Good	Excellent	Weak	No	Good	Weak
Hill number	1.12	1.79	0.66	0.90	1.39	NA	0.53	0.48
Type of channels	Type I	Type I	Type II	Type II	Type I	No	Type II	Type II
**K**^**+**^								
Nmax500	0.49	0.63	0.88	0.84	0.59	0.57	0.73	0.62
Activity	No	No	Excellent	Excellent	Weak	Weak	Good	Good
Hill number	NA	1.35	0.66	1.08	0.98	1.11	0.22	0.22
Type of channels	No	Type I	Type II	Type II	Type II	Type I	Type II	Type II
**H**^**+**^								
N_max500_	NA	NA	0.75	0.82	0.67	0.62	0.76	0.76
Activity	No	No	Excellent	Excellent	Weak	Weak	Excellent	Excellent
Hill number	NA	2.11	0.27	0.69	0.78	2.61	0.52	1.06
Type of channels	No	Type I	Type II	Type II	Type II	Type I	Type II	Type II

*N_max500_ values are the normalized maximum values of transport and n is Hill coefficient*.

**Figure 3:**
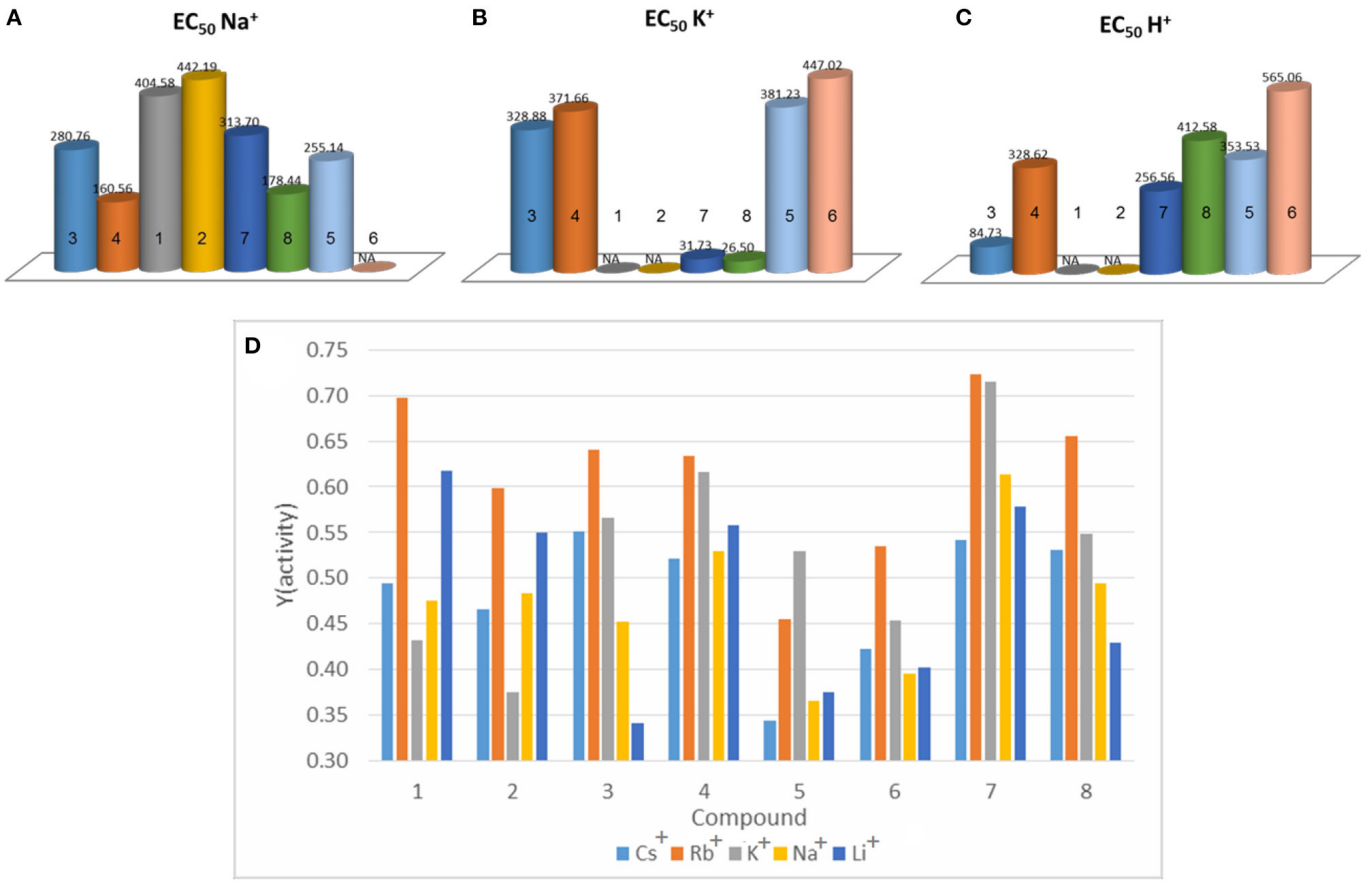
EC50 values for **(A)** Na^+^, **(B)** K^+^ and **(C)** H^+^ transport of compounds **1**-**8**. **(D)** Maximum activity of compounds **1**-**8** toward alkaline cations.

Hill analysis (Li et al., 2018) (Supplementary Figures 3, 4) revealed that the type I channels are attributed to the compounds which do not contain a fluorine substituent, while the p-substituted fluorine compounds generally favor type II channels. All the Hill coefficients are above 1, indicative of a formation of carriers (Table 1, Figures 3A–C). A Hill number >1 is specific for a type I channel. One that is lower or equal to 1 corresponds to a type II channel. For type I channels standard analyses indicate the stoichiometry of unstable supramolecular assemblies that exist besides an excess of monomer and classical structural analysis methods do not provide any useful information on the structure. In the case of type II they do, for example shifts in a proton NMR based on concentration. One last difference between the two channel types is that type II formation is exergonic, while type I formation is endergonic (Bhosale and Matile, 2006).

In the present study, one supposition is that the non-fluorinated compounds strongly self-assemble, resulting in more rigid superstructures within the membrane. The compounds containing the fluorine substituents (that probably offers higher fluidity and compatibility of the molecules within the membrane), may present a more dynamic structure and are able to form transitory channel superstructures where they accommodate ions. The EC50 values have been calculated for the active compounds. These reveal that although the overall activity is higher for compounds **3** and **4**, compounds **7** and **8** have much lower EC50 values (one order of magnitude) at the expense of 15–20% activity. Compounds **1** and **2** which underperformed in the transport of Na^+^ present no activity toward K^+^ and compounds **5** and **6** have a better affinity for K^+^ than Na^+^, but are overall very weak transporters (Figure 3).

Moreover, we were interested in further testing the compounds **1**-**8** specificity for proton transport. We thus used a classical HPTS assay and control valinomycin (K^+^ cations) and, known to generate a strong K^+^/H^+^ electrogenic antiport (Figure 2). The proton transport was monitored over a 500 second interval (Supplementary Figure 7). In the case of coupling of valinomycin with **1-8**, potential proton transport acceleration is generated. Electrogenic membrane polarization *via* Val-K^+^ transport, generates proton antiport rates through the membrane. As in the case of ionic transport the compounds containing the fluorine substituents **1**, **2** and **5**, **6** displayed higher activity than the other systems tested. The histamine compounds proved to be particularly efficient, especially the S enantiomers (Table 1). The Hill interpretation reveals that compounds **1** and **2** present no activity, while compounds **5** and **6** present a very weak activity. The minimal activity of compounds **5** and **6** is not due to the amplitude of the signal, but to the lack of correlation between concentration and activity. Consistent with the ions transport tests, the best transporters present type II channels while compounds **2** and **6** present type I channels as seen in Table 1. Compounds **4** and **5** display the best activity toward H^+^. In this case the EC50 values are this time correlated directly with the activity. Namely, the critical dose for the best transporters is also the lowest critical concentration in this case. This observation is in contrast from the K^+^ transport experiments, where the best transporters did not display the lowest EC50 values.

In the case of self-assembled artificial ion channels, selectivity toward ions is one key feature. Each type of channel, based on its structure and disposition, favors the selective transport of ions, closely tied to the dehydration energy, volume and charge of the transported ions. We have tested our library toward the transport of the monovalent alkali cations. For this reason, compounds **1**-**8** were injected in LUV solutions containing a 100 mM LiCl, NaCl, KCl, RbCl or CsCl solution. An intermediary concentration (400 μM) was chosen to avoid both capping effects and extreme behaviors dependent on high concentrations of active system, thus obtaining sufficient transport for reliable data. For each experiment a blank injection of DMSO was also recorded (Supplementary Figure 5).

The experimental dose-response curves were fitted using a Hill sigmoidal curve. This method was successfully applied to all curves with a few exceptions. In the case of compound **1** for the Rb^+^ ion, the fit satisfies the mathematical criteria, however the result has no physical sense (Y larger than 1). For some other particular cases the fit suggested a much higher activity value than the one experimentally found. As a general observation, the calculated values are similar to the experimental ones (Supplementary Tables 2, 3). By comparing the two values (Supplementary Figure 6), the calculated one, based on the initial rate of transport, and the experimental one we can differentiate between ideal and practical conditions, concluding that the read experimental activity is validated.

For the active fluorine-substituted compounds **3**, **4**, **7** and **8**, as well as the less active systems, compounds **1**, **2**, **5** and **6**, the selectivity order is Rb^+^ > K^+^ ≥ Cs^+^ > Na^+^ ≥ Li^+^. A precise selectivity order cannot be fully established if all the library is taken into account; for example, histamine-based compounds **1**-**4** present a strong activity toward Li^+^, which is not true for the triazole based compounds **5**-**8**. These differences in behavior can be attributed to the adaptive types of channels that the compounds form. In the case of fluorine substituted compounds **3**, **4**, **7** and **8**, which were proven to be type II channels by the Na^+^ and K^+^ transport, we can attribute a self-assembled labile and adaptive structure. Since these don't have a fixed number of molecules forming the channels, they are better at accommodating larger ions like Rb^+^, Cs^+^ and K^+^. In the case of the other compounds, the supposedly rigid structures, better accommodate the smaller Li^+^. Their overall lower activity, besides underlining once again the fact that they are weak transporters, also covers some selectivity differences toward certain cations.

Although each compound presents preferences toward certain cations the term selectivity is a bit restrictive in this case. Indeed, one could make a general observation, that there is a preferential activity toward the Rb^+^ and K^+^ cations (Figure 3D). The self-assembled structures described here, are very intriguing transporting systems, presenting a remarkable combination of functions influencing cation and proton transport activities. Specifically, we have demonstrated that simple structural variations of the hydrophobic part of compounds by inclusion of fluorine atoms as well as a specific chirality would strongly influence the transport activities of ions.

The multivalent self-assembly of active transporting species presented here, illustrate that “supramolecular polymorphism” can be associated with the formation of active structures within bilayer membranes. We therefore equate that a dynamic ion binding sites distribution along such directional networks, can influence the molecular-scale hydrodynamics of hydrated ions presenting variable membrane affinities and may provide inspiration for the systematic rationalization of active ion-channels.

## Author Contributions

MB: conceptualization, supervision, and funding acquisition. EL: synthesis, cation transport. AVL: investigation X-ray structures. I-MA and MB: writing—draft preparation. All authors contributed to the article and approved the submitted version.

## Conflict of Interest

The authors declare that the research was conducted in the absence of any commercial or financial relationships that could be construed as a potential conflict of interest.
