# Supplementary material for: Self-Assembled H-Bonding Superstructures for Alkali Cation and Proton Transport
Source: Front Chem. 2021 May 6;9:678962. doi: 10.3389/fchem.2021.678962 (PMC8134729; doi:10.3389/fchem.2021.678962)
Supplement: Supplementary file 1 [file Data_Sheet_1.PDF]

Supplementary Material for

Self-assembled H-bonding superstructures for alkali cation and proton transport

Erol Licsandru, Iuliana-Marilena Andrei, Arie Van der Lee, Mihail Barboiu*

Institut Europeen des Membranes, University of Montpellier, ENSCM-CNRS, Place E. Bataillon CC047, Montpellier, F-34095, France

Correspondence: mihail-dumitru.barboiu@umontpellier.fr*

Synthetic protocol: All of the compounds have been synthesized following either the scheme 1 for compounds 1-8. The amine (30 mmol) is mixed with the corresponding amount of isocyanate, under sonication (1 eq.: 1eq). The mixture was solubilized in 10 ml of THF (tetrahydrofuran), 5 ml of ethylacetate, and 10 ml of dimethylacetamide. The reaction mixture was heated to 80°C for 15 minutes. When the precipitation begins 5 ml of acetonitrile are added and the heating is maintained for another 4 hours. The resulting product will be a white powder which is then filtered and washed with methanol on the filter paper. The exact amounts of compounds used and the yields obtained are presented for a reaction between 30 mmols of each component in table S1:

Supplementary Table S1: Amounts and yields for a synthesis of compounds 1-8

	Isocyanate / formula	Isocyanate mass (g)	Amine / formula	Amine mass (g)	Overall yield (%)
1	(R)-(1-isocyanatoethyl)benzene/ C9H9NO	4.41	Histamine/ C5H9N3	3.35	91
2	(S)-(1-isocyanatoethyl)benzene/ C9H9NO	4.41	Histamine/ C5H9N3	3.35	94
3	(R)-1-fluoro-4-(1-isocyanatoethyl)benzene/ C9H8FNO	4.95	Histamine/ C5H9N3	3.35	92
4	(S)-1-fluoro-4-(1-isocyanatoethyl)benzene/ C9H8FNO	4.95	Histamine/ C5H9N3	3.35	93
5	(R)-(1-isocyanatoethyl)benzene/ C9H9NO	4.41	3-amino-1,2,4-	2.52	88
6	(S)-(1-isocyanatoethyl)benzene/ C9H9NO	4.41	3-amino-1,2,4-triazole	2.52	91
7	(R)-1-fluoro-4-(1-isocyanatoethyl)benzene/ C9H8FNO	4.95	3-amino-1,2,4-triazole	2.52	90
8	(S)-1-fluoro-4-(1-isocyanatoethyl)benzene/ C9H8FNO	4.95	3-amino-1,2,4-triazole	2.52	91

Characterization of compounds: Materials and Methods: R)-(-)-benzyl isocyanate, (S)-(+)-2-benzyl isocyanate, (R)-(-)4-Fluoro-benzyl isocyanate (S)-(-)4-Fluoro-benzyl isocyanate and histamine and 3-amino-triazole were purchased from Sigma Aldrich, 8-hydroxypyrene-1,3,6-trisulfonic acid trisodium salt (HPTS) abd Valinomicin was purchased from Fluka, , EYPC was purchased from Avanti Polar Lipids and used as received. ^{1}H NMR spectra were recorded on an ARX 300 MHz Bruker. Chemical shifts are reported as δ values (ppm) with corresponding deuterated solvent peak as an internal standard. Mass spectrometric analysis was performed in the positive ion mode using a quadrupole mass spectrometer (Micromass, Platform II). Fluorescence spectra were recorded in Perkin Elmer LS-55. X-ray single crystal data were collected on a Rigaku-Oxford Diffraction Gemini-S diffractometer (λ=0.7107 Å).The CrysAlis-Pro was used for the data collection and reduction. The Superflip program was used to solve the structures and the CRYSTALS program was used to refine the structures. All atomic displacement ellipsoids appeared to behave well.

CCDC 2069393-2069396 contain the supplementary crystallographic data for this paper. These data can be obtained free of charge from The Cambridge Crystallographic Data Centre via www.ccdc.cam.ac.uk/structures

Compound 1: (R)-1-(2-(1H-imidazol-4-yl)ethyl)-3-(1-phenylethyl)urea, ^{1}H-RMN (DMSO-d6, 300 MHz) δ (ppm) =1.29 (d,3H, *CH3*); 2,57 (t, 2H, NHCH2*CH2*); 3,21 (q, 2H, CH2*CH2*NH); 4.70 (qv, 1H, CH3*CH*NH); 5,79 (s mod, 1H, *NH*CH2); 6,37 (d mod,1H,*NH*-CH-Ph); 6,77 (s, 1H, C *CH*NH imidazole); 7.17-7.33 (m,5H, phenyl); 7,56 (s, 1H, N*CH*NH imidazole). ESI-MS: M^{*+}=259.1

Compound 2: (S)-1-(2-(1H-imidazol-4-yl)ethyl)-3-(1-phenylethyl)urea, ^{1}H-RMN (DMSO-d6, 300 MHz) δ (ppm) =1.29 (d,3H, *CH3*) ; 2,60 (t, 2H, NHCH2*CH2*) ; 3,19 (q, 2H, CH2*CH2*NH) ; 4.72 (qv, 1H, CH3*CH*NH) ; 5,79 (s mod, 1H, *NH*CH2) ; 6,37 (d mod, 1H, *NH*-CH-Ph) ; 6,80 (s, 1H, C*CH*NH imidazole) ; 7.19-7.33 (m,5H, phenyl) ; 7,61 (s, 1H, N*CH*NH imidazole), ESI-MS: M^{*+}=259.1.

Compound 3: (R)-1-(2-(1H-imidazol-4-yl)ethyl)-3-(1-(4-fluorophenyl)ethyl)urea:^{1}H-RMN (DMSO-d6, 300 MHz) δ (ppm) =1.28 (d,3H, *CH3*) ; 2,58 (t, 2H, NHCH2*CH2*) ; 3,20 (q, 2H, CH2*CH2*NH) ; 4.78 (qv, 1H, CH3*CH*NH) ; 5,80 (s mod, 1H, *NH*CH2) ; 6,40 (d mod,1H,*NH*-CH-Ph); 6,80 (s,1H,C *CH*NH imidazole) ; 7.13-7.18 (m,2H, CHCCH phenyl) ; 7.27-7.32 (m,2H,*CH*CF*CH* phenyl) ; 7,52 (s,1H,N *CH*NH imidazole). ESI MS M^{*+}=277.1

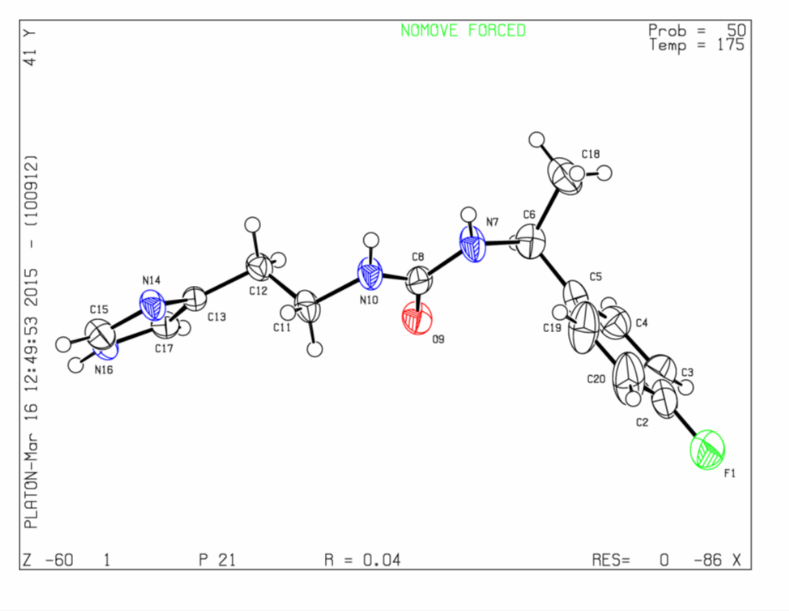

Crystallographic table	
compound	3
chemsum	C14H17FN4O
moiety	C14H17FN4O
SG	P21
a	9.1866(12)
b	5.6564(6)
c	14.2700(16)
alpha	90
beta	107.802(14)
gamma	90
volume	706.01(8)
Z	2
size	0.010x0.030x0.100
density	1.300
resol	0.86
nreftot	1756
nrefls	820
Rint	0.036
sigmaloverI	0.0651
npar	190
R1	0.0426
wR2	0.0401
GOF	1.1798

Compound 4: (S)-1-(2-(1H-imidazol-4-yl)ethyl)-3-(1-(4-fluorophenyl)ethyl)urea: ^{1}H-RMN (DMSO-d6, 300 MHz) δ (ppm) =1.28 (d, 3H, *CH3*) ; 2,58 (t, 2H, NHCH2*CH2*); 3,22 (q, 2H, CH2*CH2*NH); 4.72 (qv, 1H, CH3*CH*NH); 5,79 (s mod, 1H, *NH*CH2); 6,39 (d mod, 1H, *NH*-CH-Ph); 6,79 (s, 1H, C *CH*NH imidazole); 7.09-7.15 (m, 2H, CHCCH phenyl); 7.27-7.31 (m, 2H, *CH*CF*CH* phenyl); 7,58 (s,1H,N *CH*NH imidazole). ESI-MS: M^{*+}=277.1

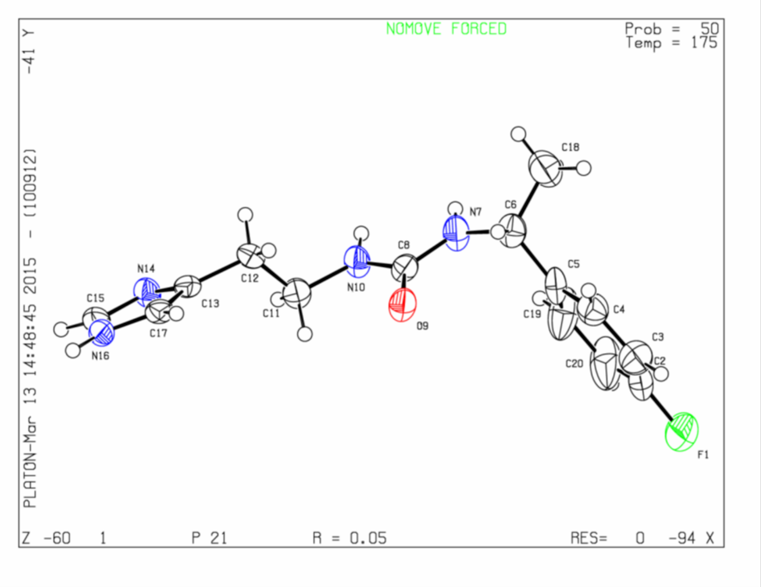

Crystallographic table	
compound	4
chemsum	C14H17FN4O
moiety	C14H17FN4O
SG	P21
a	9.181(2)
b	5.6412(8)
c	14.210(3)
alpha	90
beta	107.80(2)
gamma	90
volume	700.75(13)
Z	2
size	0.020x0.040x0.120
density	1.309
resol	0.85
nreftot	3446
nrefls	959
Rint	0.046
sigmaoverI	0.0604
npar	190
R1	0.0475
wR2	0.0524
GOF	1.1142

Compound 5: (R)-3-amino-N-(1-phenylethyl)-1H-1,2,4-triazole-1-carboxamide: ^{1}H-RMN (DMSO-d6, 300 MHz) δ (ppm) =1.52 (d, 3H, *CH3*); 4.98 (qv, 1H, CH3CHNH); 7.19-7.23 (m, 1H, CH*CH*CH phenyl) ; 7.31-7.35 (m, 2H, *CH*CH*CH* phenyl) ; 7.39-7.41 (dm, 2H, *CH*C*CH* phenyl) 7,56 (s, 1H, N*CH*N triazole), 8.52-8.56 (d, 1H, CH-*NH*-C); ES M^{*+}=232.1**.**

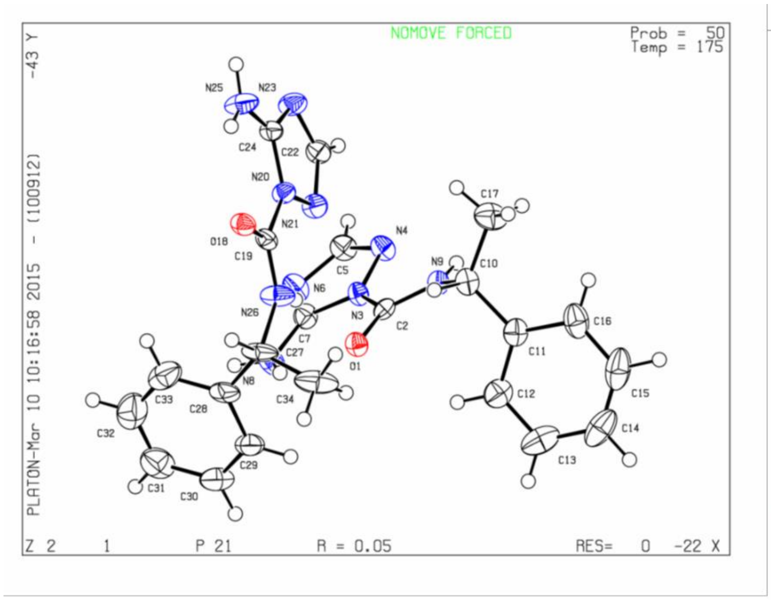

Crystallographic table	
compound	5
chemsum	C11H13N5O
moiety	C11H13N5O
SG	P21
a	8.5348(4)
b	14.7899(6)
c	9.8094(4)
alpha	90
beta	105.886(4)
gamma	90
volume	1190.94(5)
Z	4
size	0.060x0.120x0.230
density	1.290
resol	0.73
nreftot	5537
nrefls	2457
Rint	0.035
sigmaoverI	0.0509
npar	325
R1	0.0465
wR2	0.0464
GOF	1.1215

Compound 6: (S)-3-amino-N-(1-phenylethyl)-1H-1,2,4-triazole-1-carboxamide: ^{1}H-RMN (DMSO-d6, 300 MHz) δ (ppm) =1.51 (d, 3H, *CH3*) ; 4.96 (qv, 1H, CH3CHNH); 7.21-7.26 (m, 1H, CH*CH*CH phenyl) ; 7.30-7.35 (m, 2H, *CH*CH*CH* phenyl) ; 7.40-7.42 (dm, 2H, *CH*C*CH* phenyl) 7,57 (s, 1H, N*CH*N triazole), 8.52-8.58 (d, 1H, CH-*NH*-C) ESI-MSM^{*+}=232.1

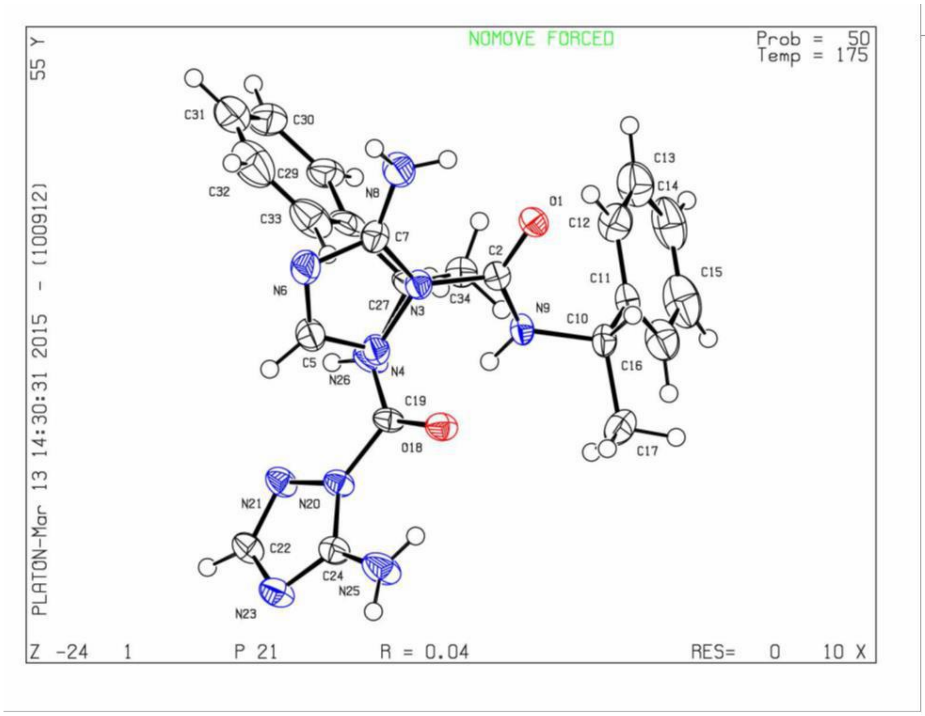

Crystallographic table	
compound	6
chemsum	C11H13N5O
moiety	C11H13N5O
SG	P21
a	8.5277(7)
b	14.8001(11)
c	9.8121(6)
alpha	90
beta	105.843(8)
gamma	90
volume	1191.34(8)
Z	4
size	0.200x0.300x0.650
density	1.289
resol	0.73
nreftot	6914
nrefls	2469
Rint	0.028
sigmaoverI	0.0422
npar	325
R1	0.0396
wR2	0.0456
GOF	1.1117

Compound 7: (R)-3-amino-N-(1-(4-fluorophenyl)ethyl)-1H-1,2,4-triazole-1-carboxamide, ^{1}H-RMN (DMSO-d6, 300 MHz) δ (ppm) =1.48 (d,3H, *CH3*) ; 4.98 (qv, 1H, CH3CHNH); 7.11-7.16 (m, 2H, CHCCH phenyl) ; 7.45-7.50 (m, 2H, *CH*CF*CH* phenyl) ; 7,57 (s, 1H, N *CH*N triazole), 8.61-8.64 (d, 1H, CH-*NH*-C) ESI M^{*+}=250.1.

Compound 8 :(S)-3-amino-N-(1-(4-fluorophenyl)ethyl)-1H-1,2,4-triazole-1-carboxamide: ^{1}H-RMN (DMSO-d6, 300 MHz) δ (ppm) =1.50 (d, 3H, *CH3*) ; 4.96 (qv, 1H, CH3CHNH); 7.12-7.18 (m, 2H, CHCCH phenyl) ; 7.43-7.48 (m, 2H, *CH*CF*CH* phenyl) ; 7,58 (s, 1H, N*CH*N triazole), 8.60-8.62 (d, 1H, CH-*NH*-C), ESI-MS M^{*+}=250.1.

Crystal structure data

	3	4	5	6
**formula**	$C_{14}H_{17}FN_4O$	$C_{14}H_{17}FN_4O$	$C_{11}H_{13}N_5O$	$C_{11}H_{13}N_5O$
**moiety**	$C_{14}H_{17}FN_4O$	$C_{14}H_{17}FN_4O$	$C_{11}H_{13}N_5O$	$C_{11}H_{13}N_5O$
***T* (K)**	175	175	175	175
**spacegroup**	$P2_1$	$P2_1$	$P2_1$	$P2_1$
**crystal system**	monoclinic	monoclinic	monoclinic	monoclinic
***a* (Å)**	9.1866(12)	9.181(2)	8.5348(4)	8.5277(7)
***b* (Å)**	5.6564(6)	5.6412(8)	14.7899(6)	14.8001(11)
***c* (Å)**	14.2700(16)	14.210(3)	9.8094(4)	9.8121(6)
***a* (º)**	90	90	90	90
***b* (º)**	107.802(14)	107.80(2)	105.886(4)	105.843(8)
***g* (º)**	90	90	90	90
***V* (Å^3)**	706.01(8)	700.75(13)	1190.94(5)	1191.34(8)
Z	2	2	4	4
***r* (gcm^{-3})**	1.300	1.309	1.290	1.289
**M_r (gmol^{-1})**	276.32	276.32	231.26	231.26
***m* (mm^{-1})**	0.095	0.095	0.089	0.089
**R_{int}**	0.036	0.046	0.035	0.028
**Q_{max} (º)**	24.526	24.854	28.993	29.258
**resolution (Å)**	0.86	0.85	0.73	0.73
**N_{tot} (measured)**	1756	3446	5537	6914
**N_{ref} (unique)**	967	1117	2797	2879
**N_{ref} (I>2s(I))**	820	959	2457	2469
**N_{ref} (least-squares)**	820	959	2457	2469
**N_{par}**	190	190	325	325
**<s(I)/I>**	0.0651	0.0604	0.0509	0.0422
**R_1 (I>2s(I))**	0.0426	0.0475	0.0465	0.0396
**wR_2 (I>2s(I))**	0.0401	0.0524	0.0464	0.0456
**R_1 (all)**	0.0551	0.0604	0.0558	0.0498
**wR_2 (all)**	0.0401	0.0506	0.0524	0.0578
**GOF**	1.1798	1.1142	1.1215	1.1117
**Dr (eÅ^{-3})**	-0.19/0.18	-0.29/0.20	-0.22/0.25	-0.20/0.19
**crystal size (mm^3)**	0.01x0.03x0.10	0.02x0.04x0.12	0.06x0.12x0.23	0.20x0.30x0.65

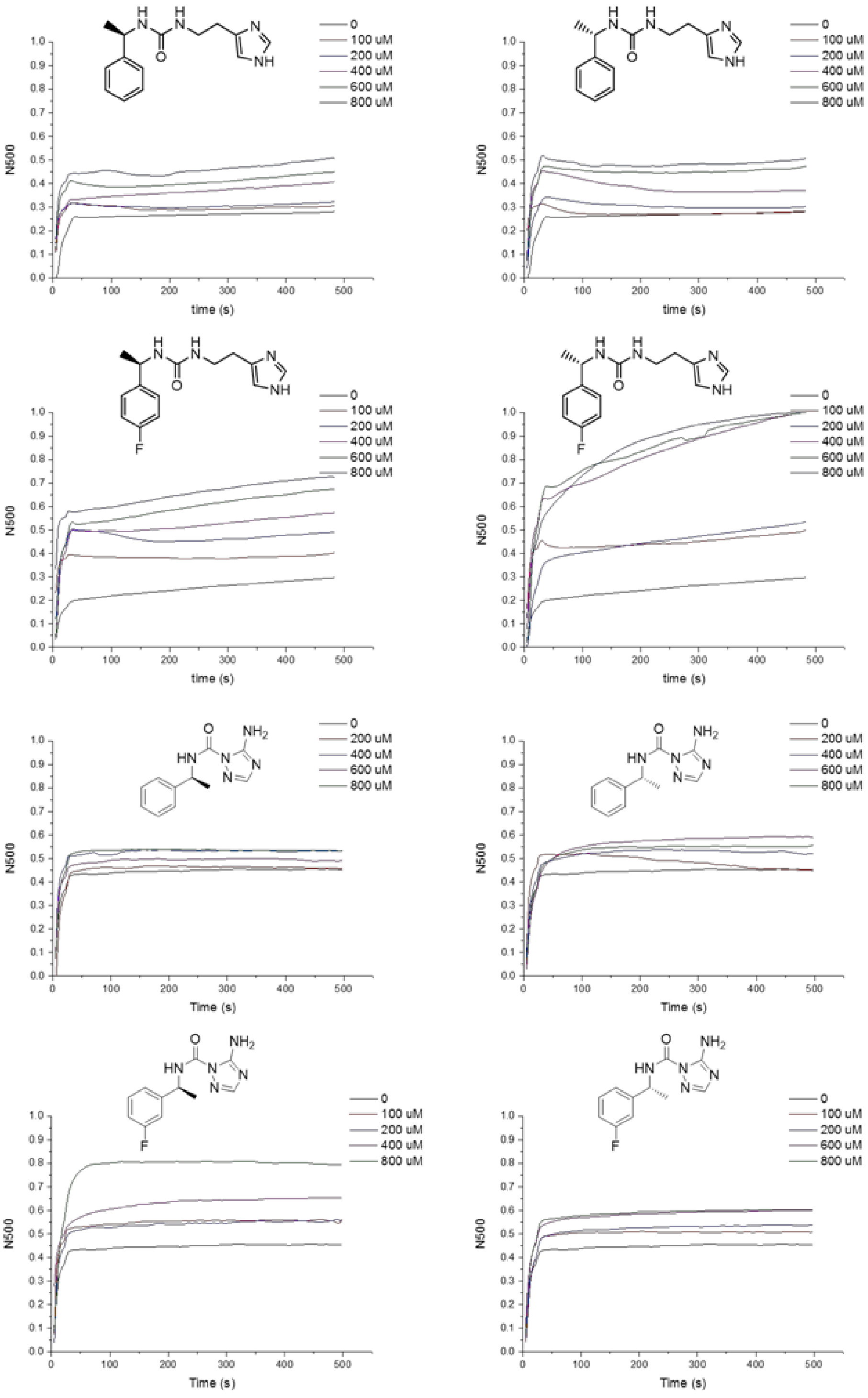

Supplementary Figure S1. Ratiometric normalized fluorescence Na^+ transport curves of compounds **1-8**. LUV suspension in PBS pH=6.4 (10mM), NaCl 100mM. N500 normalized transport over 500 seconds. Compound insertion at time=-50s, NaOH (25 µl, 0,5M) added at t=0. Fluorescent probe HPTS λ_{1ex}=405nm, λ_{2ex}=460nm, λ_{em}=510nm.

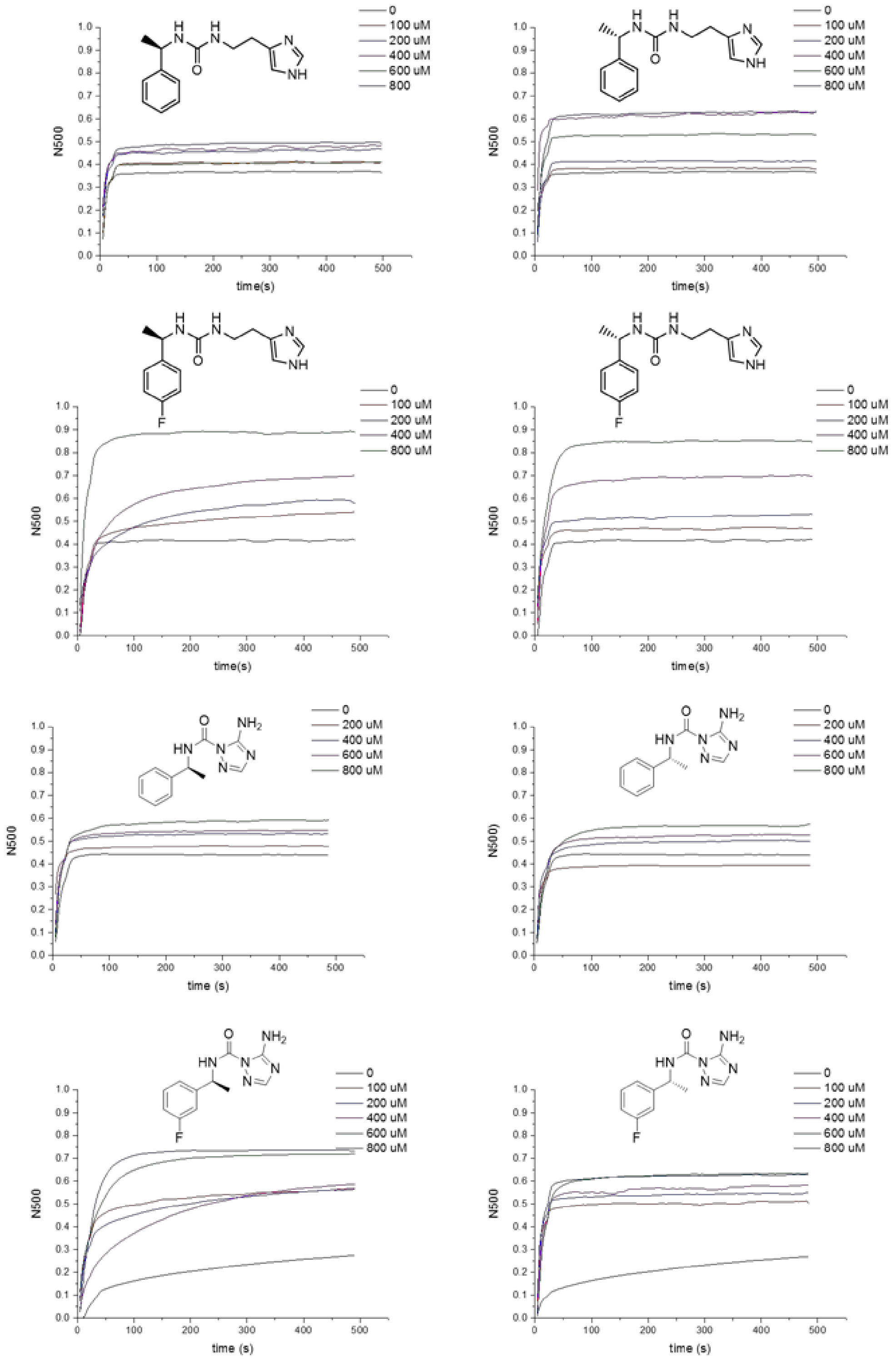

Supplementary Figure S2. Ratiometric normalized fluorescence K+ transport curves of compounds 1-8. LUV suspension in PBS pH=6.4 (10mM), KCl 100mM. N500 normalized transport over 500 seconds. Compound insertion at time=-50s, NaOH (25 µl, 0,5M) added at t=0. Fluorescent probe HPTS λ1ex=405nm, λ2ex=460nm, λem=510nm.

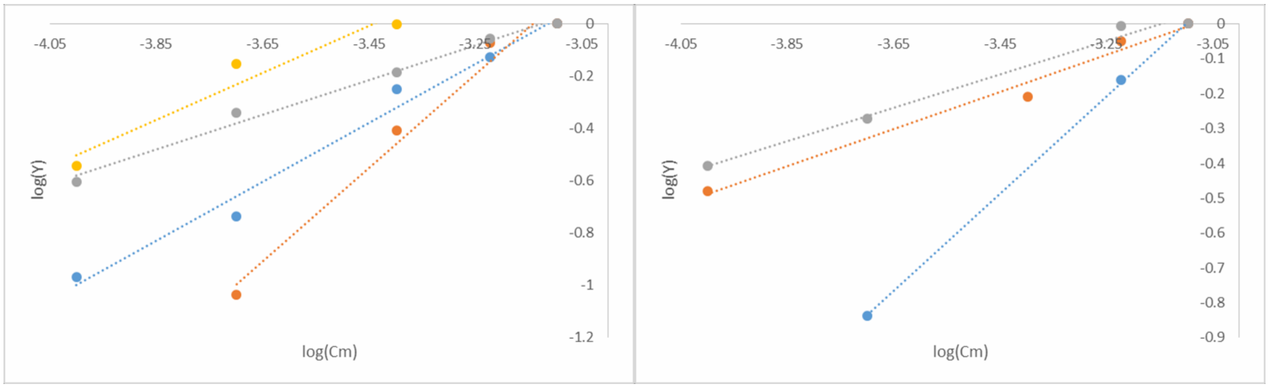

Supplementary Figure S3. Hill plots for Na^+ transport. (Left) Blue – compound 1; Red – compound 2; Gray – compound 3; Yellow – compound 4. (Right) Blue – compound 5; Red – compound 7; Gray – compound 8

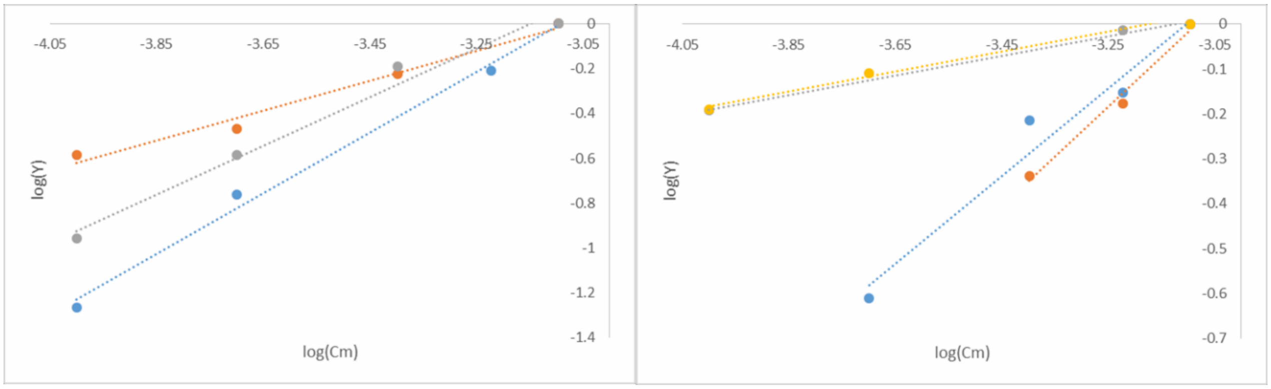

Supplementary Figure S4. Hill plots for K^+ transport. (Left) Blue – compound 2; Red – compound 3; Gray – compound 4. (Right) Blue – compound 5; Red – compound 6; Gray – compound 7; Yellow – compound 8

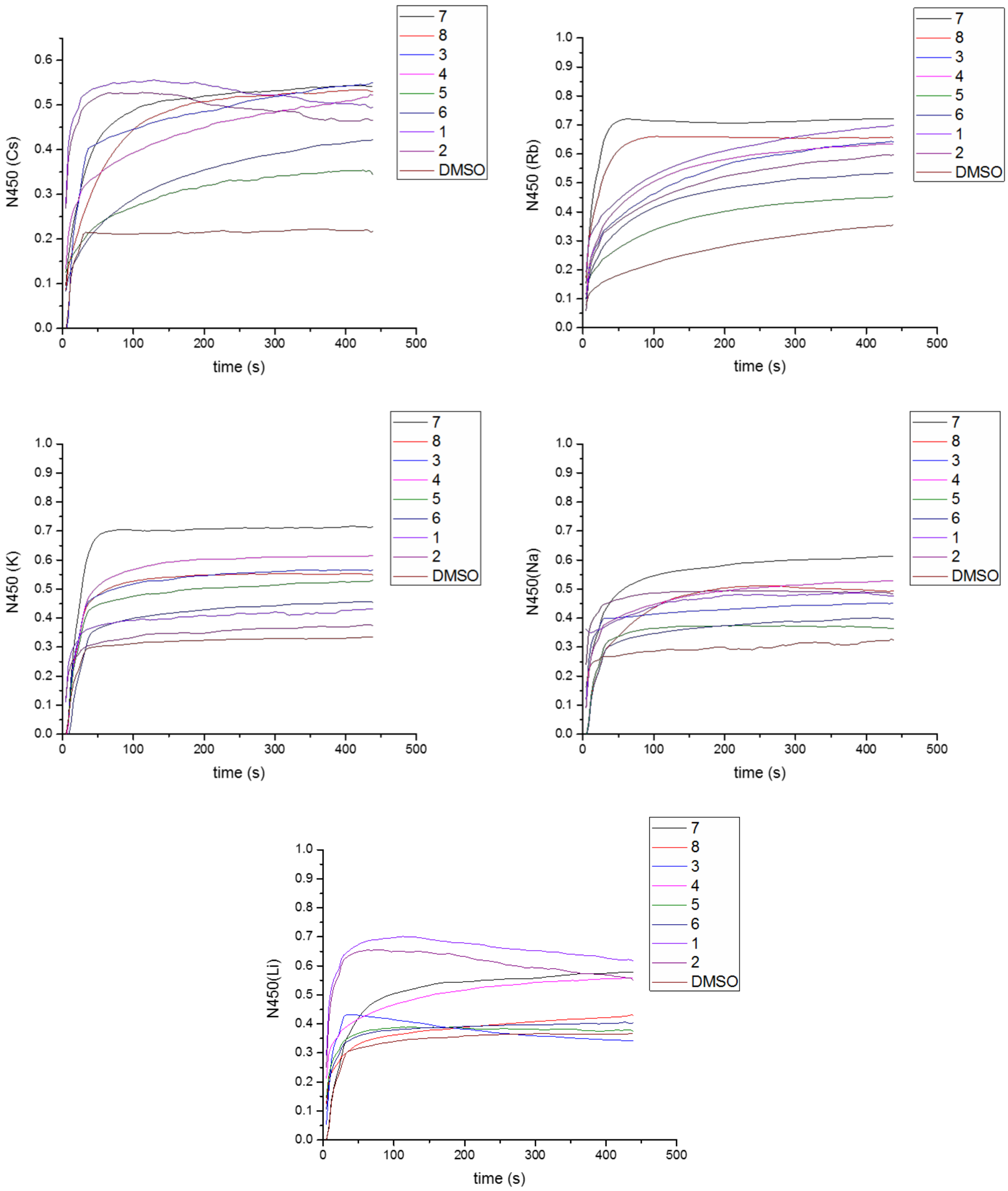

Supplementary Figure S5. Ratiometric normalized fluorescence transport curves of compounds 1-8 towards first group cations at a fixed concentration of 400 µM. LUV suspension in PBS pH=6.4 (10mM), for each transport experiment a solution of 100 mM the corresponding chloride of the transported cation was used. N450 normalized transport over 450 seconds. Compound insertion at time=-50s, NaOH (25 µl, 0,5M) added at t=0. Fluorescent probe HPTS λ1ex=405nm, λ2ex=460nm, λem=510nm.

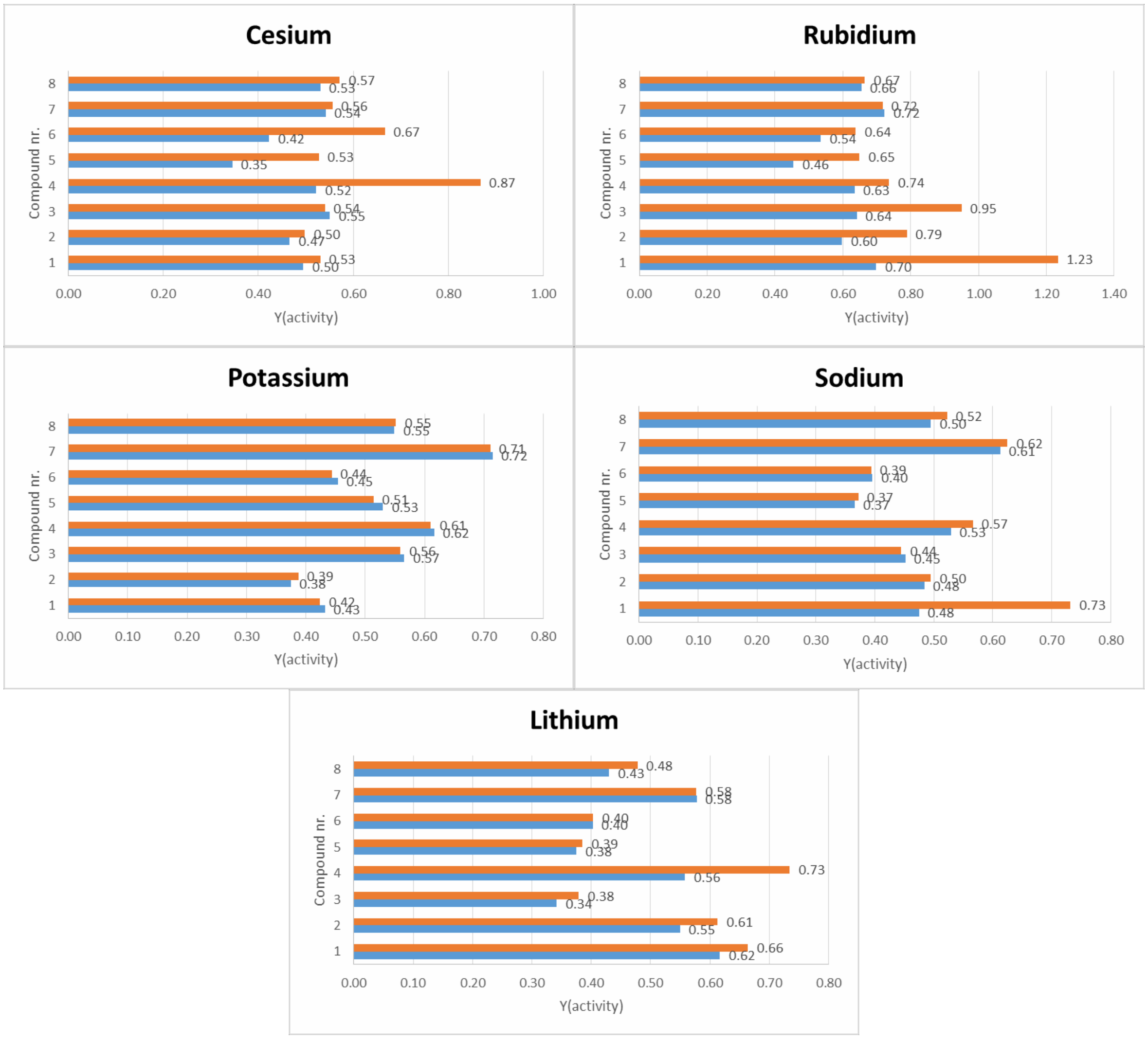

Supplementary Figure S6. Correlation between the experimental values of transport (blue) and the fitted ones (red).

Supplementary Table S2: Experimental maximum values of activity

**Comp./Ymax**	**1**	**2**	**3**	**4**	**5**	**6**	**7**	**8**
Cs^+	0495	0.466	0.551	0.522	0.345	0.423	0.542	0.531
Rb^+	0.698	0.598	0.641	0.634	0.455	0.535	0.723	0.655
K^+	0.432	0.375	0.566	0.616	0.530	0.454	0.715	0.549
Na^+	0.475	0.484	0.452	0.529	0.366	0.396	0.613	0.495
Li^+	0.617	0.550	0.342	0.558	0.375	0.403	0.578	0.430

Supplementary Table S3: Calculated maximum values of activity

Comp.	1	2	3	4	5	6	7	8
Cs^+								
Vmax	0.530	0.497	0.540	0.867	0.527	0.666	0.556	0.571
k	4.386	4.431	22.267	159.048	82.322	160.405	19.341	33.131
n	2.017	2.069	1.187	0.373	0.452	0.559	1.170	1.097
Rb^+								
Vmax	1.233	0.789	0.949	0.736	0.648	0.637	0.717	0.665
k	NA	NA	NA	NA	NA	NA	NA	NA
n	NA	NA	NA	NA	NA	NA	NA	NA
K^+								
Vmax	0.423	0.387	0.559	0.610	0.514	0.443	0.710	0.551
k	7.279	7.146	18.42	21.173	18.511	25.217	18.388	18.355
n	1.037	0.728	1.758	1.879	1.781	1.913	2.729	1.982
Na^+								
Vmax	0.731	0.495	0.444	0.566	0.371	0.393	0.624	0.522
k	17.408	4.434	6.777	15.687	16.333	20.033	16.064	29.915
n	0.227	1.191	1.136	0.761	2.213	1.442	1.070	1.417
Li^+								
Vmax	0.664	0.612	0.379	0.734	0.385	0.403	0.576	0.478
k	NA	NA	NA	NA	NA	NA	NA	NA
n	1.939	2.347	3.660	0.411	1.465	1.193	1.489	0.577

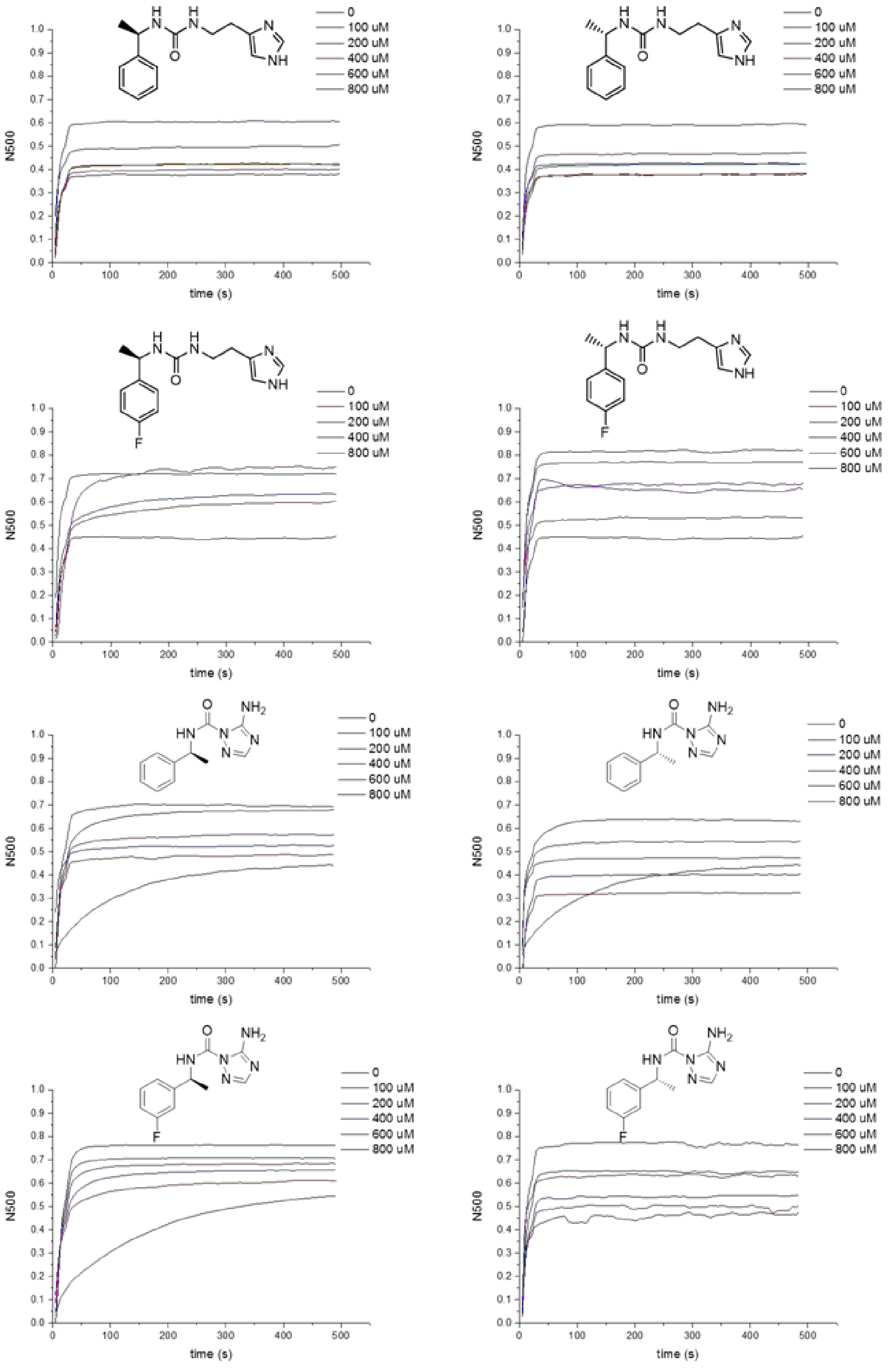

Supplementary Figure S7. Ratiometric normalized fluorescence transport curves of compounds 1-8. LUV suspension in PBS pH=6.4 (10mM), KCl 100mM. N500 normalized transport over 500 seconds. Compound insertion at time=-100s, valinomycin (1nM) insertion at time=-50s, NaOH (25 µl, 0,5M) added at t=0. Fluorescent probe HPTS λ1ex=405nm, λ2ex=460nm, λem=510nm.
